# Recent Advances of Cell Membrane Coated Nanoparticles in Treating Cardiovascular Disorders

**DOI:** 10.3390/molecules26113428

**Published:** 2021-06-05

**Authors:** Chaojie Zhu, Junkai Ma, Zhiheng Ji, Jie Shen, Qiwen Wang

**Affiliations:** 1 Department of Cardiology, The First Affiliated Hospital, Zhejiang University School of Medicine, Hangzhou 310003, China; 3180101530@zju.edu.cn; 2 Chu Kochen Honors College, Zhejiang University, Hangzhou 310058, China; 3180101531@zju.edu.cn (J.M.); 3180103158@zju.edu.cn (Z.J.); 3 Institute of Pharmaceutics, College of Pharmaceutical Sciences, Zhejiang University, Hangzhou 310058, China; 4 Department of Pharmacy, School of Medicine, Zhejiang University City College, Hangzhou 310015, China

**Keywords:** cell membrane coated nanoparticle, atherosclerosis, thrombosis, diagnosis and therapy, cardiovascular disease

## Abstract

Cardiovascular diseases (CVDs) are the leading cause of death worldwide, causing approximately 17.9 million deaths annually, an estimated 31% of all deaths, according to the WHO. CVDs are essentially rooted in atherosclerosis and are clinically classified into coronary heart disease, stroke and peripheral vascular disorders. Current clinical interventions include early diagnosis, the insertion of stents, and long-term preventive therapy. However, clinical diagnostic and therapeutic tools are subject to a number of limitations including, but not limited to, potential toxicity induced by contrast agents and unexpected bleeding caused by anti-platelet drugs. Nanomedicine has achieved great advancements in biomedical area. Among them, cell membrane coated nanoparticles, denoted as CMCNPs, have acquired enormous expectations due to their biomimetic properties. Such membrane coating technology not only helps avoid immune clearance, but also endows nanoparticles with diverse cellular and functional mimicry. In this review, we will describe the superiorities of CMCNPs in treating cardiovascular diseases and their potentials in optimizing current clinical managements.

## 1. Introduction

Cardiovascular disease (CVD) surpasses cancer as the most common cause of mortality [1], contributing to almost 40% total deaths in China [2]. Conventional therapeutic options include medications embodying anticoagulants [3], antiplatelet [4], thrombolytic [5] and antilipemic agents [6], and surgery including vessel bypass grafting [7] and stent insertion [8,9]. Nonetheless, disease reoccurrences, which have been reported to be 50% for any CVD event or subsequent revascularization in the year after myocardial infarction [10], side effects, for example bleeding events (occurring in around 1-8%) induced by dual antiplatelet therapy in treating acute coronary heart syndrome [11], and a high frequency of adverse drug reactions (~20%) [12], remain challenging. This is especially true for small-molecule based agents, which are organic compounds influencing molecule pathways by targeting vital functional proteins displayed on blood vessels and the heart; off-target toxicities, systemic degradation, short half-life and low bioavailability hinder clinical treatment [13,14,15].

Nanotechnology has achieved great advancements in the biomedical field. Nanomaterials facilitate targeted small-molecule drug delivery to the specific lesion site, or they may sometimes perform as a pharmacological active compound per se due to their unique physical and chemical properties [16]. Nevertheless, without any surface modifications, these nanoplatforms are rapidly removed from circulation into the liver and spleen by the body’s reticuloendothelial system (RES), which severely hampers their therapeutic efficacy [17]. Nanomaterials surface PEGylation is the most extensive measurement taken to improve their biocompatibility and prolong the circulation time [18]. However, such a method has been associated with the potential toxicity effect of inducing hypersensitivity reactions which can provoke an anaphylactic shock [19]. Alternatively, cell membrane coating technology has been reported to elicit immune evasion and prolong nanocarriers’ circulation time. Furthermore, the functional proteins on the cloaked cell membranes render additional biological properties for nanoparticles, such as selective adherence, inflammatory site targeting and endothelium penetration [20]. In this review, we briefly introduce the pathogenesis, therapeutic targets and current clinical medications of several main cardiovascular diseases. Then, we will explain fundamental information about cell membrane coated nanoparticles, including their history, characteristics and synthetic routes. To elucidate the significance of CMCNPs, we describe the evolution of CVD treatments. Through highlighting the bottlenecks of clinical medications and conventional nanomedicine in chronological sequence, we demonstrate the superiority of CMCNPs in treating CVDs. In particular, we show that CMCNPs boast a number of unique therapeutic characteristics, such as their intrinsic capacity to target lesions. As far as we know, it is the first work that describes the evolution of CVDs treatments—from clinical medications, to conventional nanomedicine, and finally to CMCNPs, with a special focus on CMCNPs.

## 2. Cardiovascular Diseases

**Figure 1:**
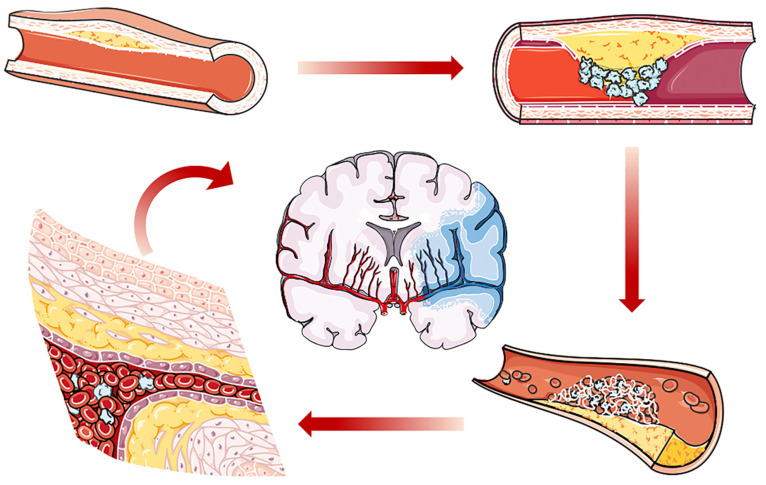
Schematic diagram showing the process of atherosclerosis. Under pathologic conditions, atherosclerosis develops with plaque formation. Atherosclerotic plaque will rupture with thrombus formation. Both thrombosis and atherosclerosis will cause local ischemia, resulting in coronary disease or peripheral arterial disease. In addition, thrombus may travel to the brain arteries, which induces ischemic stroke.

The cardiovascular system, or circulatory system, consisting of the heart and numerous vessels, is responsible for blood circulation in the body and crucial for oxygen, nutrition and cellular traffic. Behavioral risk factors such as smoking [21], hypertension [22], and diabetes [23] and biological risk factors such as age, gender [24], and family history [25], increase the prevalence of cardiovascular disease worldwide [26]. The main categories of CVDs include coronary heart disease, stroke and peripheral vascular disorders. These aforementioned CVDs are triggered by abnormal blood flow, either due to blood blockage or bleeding, the occurrence of which is essentially on account of atherosclerosis or thrombosis (Figure 1) [27]. Clinical measurements include early diagnosis, preventive care or surgical intervention. Despite these efforts to prevent and treat CVDs, progress has been moderate, and the annual total of CVD cases has seen a continuous increase in the last 30 years [28].

### 2.1. Mechanisms of Atherosclerosis and Thrombosis

#### 2.1.1. Atherosclerosis

Atherosclerosis (AS) is the most common cause of myocardial infarction and ischemia, which is featured by chronic inflammation. An intimate correlation has been reported between low-density lipoprotein (LDL), one of the five major groups of lipoprotein responsible for fat molecules transporting throughout the body in the extracellular water, and atherosclerosis [29]. Elevated levels of LDL upregulate the expression of endothelial cell adhesion molecules which can drive the infiltration of leukocytes into vessels through classical recruitment cascades [30]. Furthermore, the chemokines released from endothelial cells and activated macrophages recruit peripheral neutrophils and monocytes. In the late stage of the disease, monocytes recruited to the lesion continuously digest modified lipoprotein, differentiating into foam cells [31]. Proteases released by the apoptotic foam cells further destroy the thin endothelial cell layer which covers and protects the atherosclerotic plaque, eventually leading to plaque rupture and thrombus formation [32].

#### 2.1.2. Thrombosis

Thrombosis is defined as the formation of a blood clot within the vascular system, which severely obstructs normal blood circulation and may eventually lead to myocardial infarction and ischemic stroke. Under normal conditions, endothelial cells will actively prevent the formation of thrombus through various factors which block platelet adhesion and aggregation, inhibit coagulation and lyse the clots. Three main abnormalities of circulation facilitate thrombus formation; endothelial injury, alterations in blood flow and hypercoagulation, collectively named “Virchow’s Triad” [33]. The clot formed during this process promotes hypoxia and inflammation at the lesion site, subsequently promoting the infiltration of activated immune cells. Lesional monocytes and macrophages express metalloproteinase (MMP), which can facilitate thrombus resolution. When unresolved, clots will induce ischemia-related cardiovascular diseases, such as lower limb ischemia and ischemic stroke [34].

### 2.2. Classifications of CVDs

Cardiovascular diseases can be mainly classified into three categories—including coronary heart disease, stroke and peripheral vascular disorders—based on the location of the injury. In the next section, the main risks, manifestations, and disease mechanisms will be discussed.

#### 2.2.1. Coronary Heart Disease

Coronary heart disease, otherwise called ischemic heart disease, occurs due to insufficient blood flow to the heart and the necrosis of myocardial tissues induced by a lack of oxygen. In the early stages of the disease, a shortage of blood flow is mainly caused by atherosclerotic plaque obstruction. When the atherosclerotic plaque suddenly breaks off, it exposes its highly thrombotic components, consequently leading to thrombus formation. Thrombus formed during this process may completely obstruct the blood vessel through thrombo-embolism, which potentiates myocardial infarction [35,36].

#### 2.2.2. Stroke

Stroke will occur when the supply of blood to the brain tissue is impacted. Strokes can be divided into two subtypes—ischemic stroke and hemorrhagic stroke—based on their mechanism of formation. Hemorrhagic stroke, which makes up almost 13% of total clinical cases, is usually caused by hypertension, which results in intracranial hypertension and oxygen and nutrition depletion in the downstream tissues, rapidly inducing damage to the brain tissues [37]. On the other hand, atherosclerosis is the major cause of ischemic stroke. As a result, thrombus forms due to the local inflammation and ulceration of the fibrous plaques. The clot occludes the atherosclerotic vessel or travels further to block the brain’s arteries, causing ischemic stroke [38]. The resulting oxygen- and glucose-depleted environment downregulates junctional protein expression, which in turn facilitates the extravasation of proteins and leukocytes infiltration. Leukocytes, e.g., neutrophils, recruited to the brain lesion site during the acute inflammatory phase exacerbate inflammation through the generation of reactive oxygen species, worsening the disease condition [39,40].

#### 2.2.3. Peripheral Vascular Disorders

Peripheral vascular disorder, or peripheral arterial disease, occurs in parts of the body other than the brain and heart, such as legs and arms, the major causes of which include atherosclerosis and thrombo-embolism. Narrowing lumens induce insufficient blood flow into the lesion site, leading to local ischemia, and eventually developing into intermittent claudication. Aggravating intermittent claudication may cause critical limb ischemia, and ultimately progress into acute limb ischemia when the blood flow is completely obstructed [41].

### 2.3. Clinical Management and Its Bottleneck

Before therapeutic intervention, appropriate diagnosis should be implemented. Current clinic diagnostic tools mainly include X-ray, doppler ultrasound, CT angiography and magnetic resonance imaging angiography. However, early stage diagnosis of atherosclerosis remains a major issue due to inadequate diagnostic clarity and clinical acumen. To facilitate early diagnosis of atherosclerosis, iodinated compounds and gadolinium are two representative contrast dyes applied for CT angiography and magnetic resonance imaging angiograph, respectively. Although these measurements have achieved success to a certain degree, the biosafety of contrast agents has aroused concerns. For example, iodinated compounds are limiting in those with hypersensitivity or significant renal dysfunction. Gadolinium can also be limiting due to its potential toxicity in inducing nephrogenic systemic fibrosis, which is a rare but unmanageable scleroderma like disease [42].

Apart from diagnosis, there are also hurdles for the management and prevention of cardiovascular diseases. For high-risk patients who have not yet experienced CVDs, long-term preventive medications, such as statins, are recommended. However, a lack of patient compliance hinders our fight against CVDs [43]. In addition, anticoagulant, thrombolysis and antiplatelet agents are clinically used to treat thrombo-embolism-related CVDs. Despite the efficacy of these drugs at impeding the process of plaque development and thrombus formation, they come with a significant risk of unexpected bleeding, for example coumarin, a vitamin K antagonist, is related to a risk of major bleeding that ranges from 2% to 13% during the mean duration of follow-up of 6 to 30 months [44]. Furthermore, the therapeutic dosage varies between patients and with disease stage. Last but not least, clinical strategies against acute ischemic stroke are mainly divided into intravenous thrombolysis and endovascular thrombectomy, both of which are time-critical and have extremely limited therapeutic time windows [45]. The performance of stents also encounters bottlenecks. Bare metal stents (BMS) and drug-eluting stents (DES) are two conventional classes applied in clinical settings. DES are platforms for delivering therapeutic molecules locally, which were developed to overcome several side effects associated with BMS, such as thrombosis and neointimal hyperplasia. Although DES exhibit efficacies in preventing restenosis and scar tissue formation to some extent, in-stent thrombosis due to inflammation or certain gradient on stents, for example polymers, is the main challenge [46]. Therefore, there remains an urgent need for better alternatives with improved diagnostic and therapeutic efficacy.

## 3. Nanomedicine against CVDs

Recent advancements achieved in nanomedicine exhibit great potential to improve off-target side effects, long-term toxicity and limited diagnostic or therapeutic efficacy [47]. For example, conventional use of stents are accompanied with risks of thrombosis and restenosis. Especially for DES, late stent thrombosis (>30 days) is a main challenge. Nanoparticles have been incorporated into stents’ formulations to modulate their drug releasing properties and achieve robust endothelial healing. Liu et al. developed advanced DES, which were composed of collagen and nitric oxide (NO) donor-loaded PLGA nanoparticles [48]. As a result, such platform alleviated intima formation and exhibited a sustained release capability of NO, which significantly reduced platelet aggregation in rabbit blood, thus mitigating thrombosis.

Conventional diagnostic contrast agents, such as gadopentetic, take effect through revealing the narrowing of the vessels, termed stenosis. In contrast, nano-based molecular contrast agent could directly disclose the location of atherosclerotic plaques through active targeting [49]. For example, Qiao et al. developed an osteopontin antibody conjugated upconversion nanoplatform to achieve noninvasive targeting and imaging of vulnerable plaques [50]. Morishige et al. reported dextran coated superparamagnetic nanoparticles, which exhibited affinity towards macrophages and could be utilized to assess macrophage burden in atherosclerosis, providing a useful tool for identifying inflamed plaques and monitoring disease conditions [51].

Nanoparticles have also achieved breathtaking therapeutic improvements. The current clinical prevention of atherosclerosis mainly relies on the long term oral administration of statins, with insignificant effects unless taking an extremely high dosage [52]. However, such high dosage will inevitably induce hepatoxicity and myopathy [53]. To address this predicament, Kim et al. reported a cargo-switching nanoparticles composed of a cyclodextrin shell component and simvastatin as a loaded drug to achieve higher affinity towards atherosclerotic lesion and plaque regression [54]. In another way, Flores et al. developed single walled carbon nanotubes loaded with a chemical inhibitor of the antiphagocytic CD47-SIRPα signaling axis. Such nanoplatforms exhibited macrophage-specific targeting ability and could reactivate lesional phagocytosis to effectively reduce plaque burden [55]. Reperfusion is vital for better prognosis in acute stroke. Marsh et al. reported fibrin-targeted perfluorocarbon nanoparticles, the surface of which is modified to deliver the plasminogen activator streptokinase, a thrombolytic agent. Such a nanoplatform exhibited an improved clot-targeting ability with a lower risk of adverse hemorrhagic events [56].

However, these strategies still face the problem of fast clearance by RES. Better therapeutic or diagnostic capabilities can be obtained via prolonging their circulation. In addition, the targeting abilities of these platforms usually rely on peptide or protein (antibody) conjugation. Many factors limit their biofunctions and targeting abilities, for example, proteases in circulation in the body may interact with them, thus depriving them of their functions [57]. Alternative solutions are needed to address these drawbacks.

## 4. Cell Membrane Coated Nanoparticle and Its Applications in CVDs

Cell membrane coating technology, an alternative surface passivation method substituting for PEGylation, generally exploits innate cell membranes to coat synthetic nanoparticles. Such a coating measurement helps to disguise nanoparticles as some of the body’s intrinsic cells, which not only assists them in avoiding the body’s immune clearance, thus prolonging their circulation (higher biocompatibility), but also endows them with various cell-like biofunctions via the diverse functional membrane proteins on CMCNPs’ surface (cell-mimicking properties).

### 4.1. Cell Membrane Coated Nanoparticles

**Figure 2:**
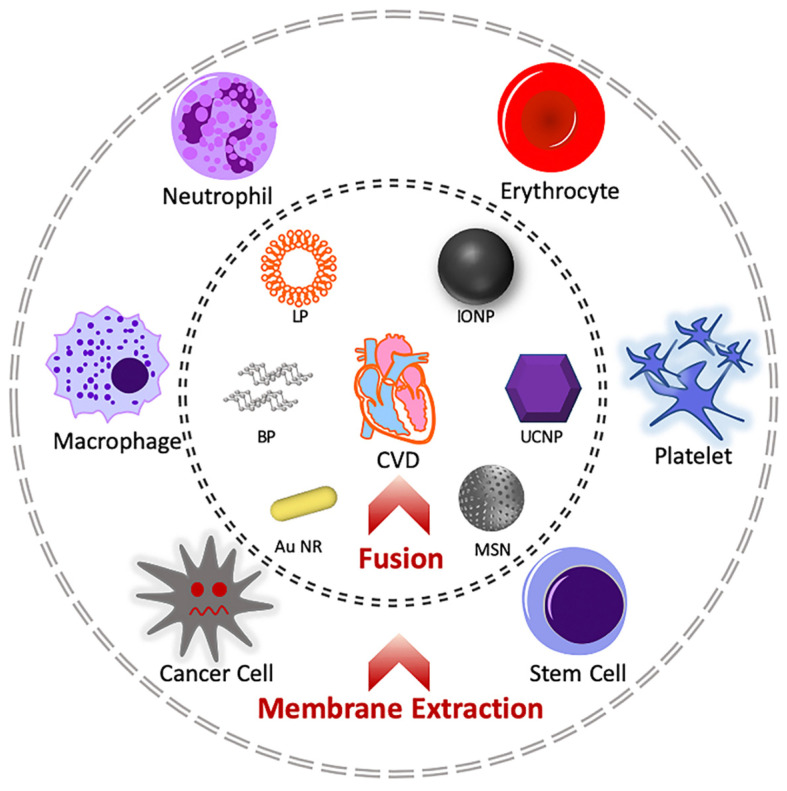
Schematic diagram showing the synthetic process of cell membrane coated nanoparticles. Basically, interested membranes are first extracted from the cell sources, e.g., macrophages, neutrophils, erythrocytes, platelets, cancer cells and stem cells. The membranes are further coated on selected nanoparticles, e.g., black phosphorous, liposome, iron oxide nanoparticle, upconversion nanoparticle, gold nanorod and mesoporous silica nanoparticle. The membrane–core hybrid system can be applied to treat cardiovascular diseases. Abbreviations: BP: black phosphorous; LP: liposome; IONP: iron oxide nanoparticle; UCNP: upconversion nanoparticle; Au NR: gold nanorod; MSN: mesoporous silica nanoparticle.

Cell membrane cloaking technology was first achieved by Hu et al. who applied red blood cells’ membranes to coat poly-(D,L-lactic-co-glycolic) acid (PLGA) nanoparticles, successfully prolonging their circulation by up to 72 h [58]. Considerable research has been conducted to expand cell membranes sources, inner nanoparticles and synthetic routes. Generally, this technique is divided into three procedures to synthesize cell membrane coated nanoparticles: cell membrane extraction, inner core nanoparticle preparation and the fusion process (Figure 2) [59].

#### 4.1.1. Cell Membrane Extraction

Cell membranes are mainly composed of phospholipids and diverse functional proteins [60]. The membrane plays a vital role in dividing cell types, cells’ intercommunications, signal transduction and cargo selective permeability. Current procedures of cell membrane extraction include membrane lysis and purification [61]. Generally, interested cells are first isolated from whole blood. Following hypotonic treatment for cell lysis, discontinuous sucrose gradient centrifugations are implemented to remove the nucleic and cytoplasmic contents. Further purifications are carried out through washing with specific buffers and extrusion through porous polycarbonate membrane to obtain the purified extracted cell membranes [62].

#### 4.1.2. Core Preparation

A number of novel nanocarriers have emerged (Table 1), e.g., liposomes [62,63], gold nanoparticles [64], mesoporous silica nanoparticles [65,66], iron oxide nanoparticles[67,68], black phosphorous [69,70,71], PLGA nanoparticles [58,72], and layered double hydroxide [73]. These nanocarriers can either act as drug delivery platforms or as active components in themselves due to their intrinsic physical and chemical properties. For example, black phosphorous 2D nanosheets can be used in photothermal therapy due to its efficient photothermal conversion property [71]. Layered double hydroxide can not only perform as lipid regulator to treat CVDs [74], but can also serve as an immune adjuvant and antigen carrier bi-functional nanoplatform in cancer immunotherapy [75]. To achieve various biological functions, e.g., magnetic resonance imaging, drug carrying or reactive oxygen species scavenging, appropriate inner nanoparticle selection is essential to fulfill the therapeutic potential of cell membrane coated nanoparticles.

#### 4.1.3. Fusion Process

The fusion process covers the inner core nanoparticles with the extracted and purified cell membranes. Generally, this process is divided into two main approaches: membrane extrusion or sonication bath. The membrane extrusion process is achieved through mixing the cell membrane vesicles and inner nanoparticles and extruding them through porous polycarbonate membranes for several repeated cycles. This method is mainly applied for small-scale production, which is suitable for laboratory use [77]. A sonication bath is a route providing higher yields but requires strictly controlled sonication power. Excessively high temperatures will denature the functional proteins on the obtained cell membrane vesicles, which severely impacts their biological performance. In addition, core–membrane nanoparticles produced through sonication methods exhibit poor size uniformity [78]. Therefore, new strategies are urgently needed with higher production efficiency, improved particle uniformity and valid membrane protein functions.

### 4.2. Cell Membrane Coated Nanoparticles in Treating Cardiovascular Disease

Cell membrane coated nanoparticles are expected to exhibit some intrinsic cell properties, e.g., specific targeting to inflammatory site, immune evasion, binding affinity to targeted receptors or cells (Table 2). In dealing with cardiovascular diseases, CMCNPs are exploited to mimic peripheral cells, such as erythrocytes [79], platelets [80], or immune cells [81], which have been reported to exhibit a crucial role in disease progression. In the following section, cell membrane coated nanoparticles will be discussed based on the context of their sources and their potential for treating CVDs.

#### 4.2.1. Erythrocytes Cell Membranes

Erythrocytes (or red-blood cells, RBC) are the most abundant cells in the human body, taking the responsibility of oxygen and carbon dioxide transport. Mechanistically, healthy erythrocytes can evade the mononuclear phagocyte system (MPS) through surface membrane protein CD47, which act as “do not eat me” signals to immune cells [91]. Therefore, erythrocytes membranes are intrinsically biocompatible and nonimmunogenic, so they can be utilized to prolong the circulation time of nanoparticles. Apart from this, RBC membrane coated nanoparticles are also explored to act as biomimetic nanosponges for detoxification [92], or serve as nanotoxoids for safe and effective toxin nanovaccination [93].

**Figure 3:**
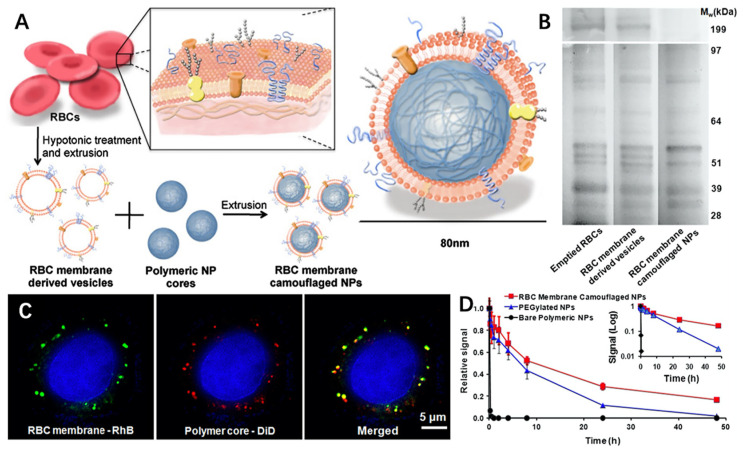
(**A**) Synthetic route of RBC-NPs; (**B**) SDS-PAGE result of emptied RBCs, RBC membrane-derived vesicles and RBC membrane camouflaged NPs; (**C**) Scanning fluorescence microscopy images of colocalization of RBC membranes (marked with green rhodamine-DMPE dyes) and polymeric cores (marked with red DiD dyes) after being internalized by HeLa cells; (**D**) Comparison of systemic circulation time between RBC-NPs, PEGylated NPs and PLGA nanoparticles. Reproduced with permission [41]. Copyright 2011, National Academy of Sciences.

As mentioned above, Zhang et al. were the first to apply erythrocyte membrane coating on PLGA nanoparticles [58]. In their study, the RBC membrane was extracted through hypotonic medium hemolysis and fused with PLGA nanoparticles to obtain RBC membrane camouflaged NPs (RBC-NPs), with an 80 nm average diameter (Figure 3A). They further proved that the RBC membrane proteins were successfully transferred onto PLGA nanoparticles (Figure 3B). Additionally, RBC membrane coated nanoparticles exhibited outstanding stability in vitro, maintaining its core-shell structure even after 6 h co-incubation with HeLa (Figure 3C). As a result, such a nanoplatform exhibits an outstanding ability to avoid immune clearance, greatly prolonging its circulation time (Figure 3D). Due to its long circulation time, this nanostructure could serve as a universally effective drug delivery platform against CVDs when suitable small-molecule agents are loaded in the inner PLGA core. For example, Wang et al. reported rapamycin-loaded PLGA nanoparticles, which were cloaked with RBC membranes (RBC/RAP@PLGA). As a result, this nanoplatform effectively attenuated the progression of atherosclerosis, in which the average area ration of plaque to vascular lumen decreased from 47.95% to 31.34% after treatment, superior to the free drug group (from 47.95% to 42.42%) [94].

Alternatively, Shao et al. prepared a Janus-type polymeric micromotors (JPMs), composed of heparin (Hep) and chitosan (CHI), coated with RBC membrane [95]. This functional nanoparticle achieved efficient motion toward the thrombus in response to near-infrared (NIR) irradiation via thermal effects and could synergize with photothermal therapy for thrombus alleviation.

The nanoplatforms discussed above mainly utilize the biggest advantage of red blood cell membrane: biocompatibility and immune evasion. Commonly, RBC membranes are coated on drug-loaded PLGA nanoparticles to achieve better drug on-target delivery against cardiovascular diseases.

#### 4.2.2. Platelet Membrane

Platelets are small anucleated blood cells engaged in the blood clotting process. Upon hemorrhage, platelets rapidly migrate to the damaged lesion to form a clot and prevent excessive bleeding. Platelets are the connectors between thrombosis, inflammation and atherosclerosis. When atherosclerotic plaque rupture, vascular injury and decelerated blood flow facilitate platelet activation and binding to the injured vascular wall, eventually leading to thrombus formation [96]. Nanoparticles coated with platelet membrane can exhibit many similar characteristics to platelets, for example, adherence to injured vasculature.

Inspired by this, Zhang et al. firstly developed platelet membrane coated PLGA nanoparticles, which exhibited selective adhesion to damaged human and rodent vasculature and MRSA252 [78]. These platelet membrane coated nanoformulations could be utilized to treat thrombus, arterial injuries and sepsis with appropriate loading agents in the inner PLGA core. For example, when dealing with thrombus, Wang et al. synthesized platelet membrane coated PLGA nanoparticles, loaded with lumbrokinase, a conventional anticoagulant agent (PNPs/LBK) [97]. As a result, PNP/LBK exhibits better thrombus targeting ability with lower hemorrhagic risks.

When thrombus occurs, insufficient blood flow lead to a hypoxic environment that promotes reactive oxygen species (ROS) generation and tissue damage in the lesion site. Inspired by this, Zhao et al. recently developed H_2_O_2_-responsive platelet membrane cloaked argatroban-loaded polymeric nanoparticles (PNPArg) to treat thrombus [98]. In their strategy, the inner core, Poly(vanillyl alcohol-co-oxalate) (PVAX), is a H_2_O_2_ degradable polymer that can scavenge excessive ROS and could synergize with argatroban, an anticoagulant agent, showing great therapeutic effects toward various thrombotic diseases.

CMCNPs can also serve as novel diagnostic tools. Ma et al. prepared a platelet membrane coated nanoconstruct (PM-PAAO-UCNPs), which consists of upconversion nanoparticles and Ce6 photosensitizer for accurate localization and non-invasive photodynamic therapy of atherosclerosis [99]. In their study, the platelet membrane coating strategy effectively increased the binding affinity of nanoparticles to foam cells, the features of which include unregulated lipid metabolism, and play a central role throughout the disease stages. Near-infrared light was then applied to induce ROS-mediated apoptosis and regulated metabolism of foam cells to treat atherosclerosis.

**Figure 4:**
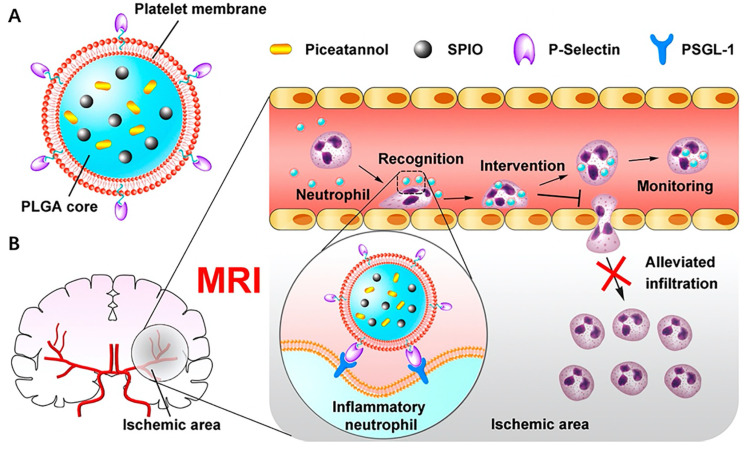
(**A**) Structure of platelet membrane coated PLGA nanoparticle; (**B**) Schematic diagram of therapeutic mechanism. Platelet membrane coated nanoparticles can straightly target the inflammatory neutrophils and take effect through releasing piceatannol to block neutrophils’ infiltration. Reproduced with permission [85]. Copyright 2019, American Chemical Society.

Apart from targeting vascular endothelial cells and foam cells, platelets also exhibit binding affinity towards activated neutrophils. During acute ischemic stroke (AIS), neutrophils migrate into cerebral ischemic regions with the aid of platelets. Recruited neutrophils release reactive oxygen species, which are the prime cause of reperfusion injury following AIS [100,101]. Studies have shown potential curative benefits through alleviating the infiltration of neutrophils [102]. Inspired by these findings, Tang et al. innovatively constructed a platelet membrane coated PLGA nanoplatform with superparamagnetic iron oxide nanoparticles and piceatannol loaded (PTNPs) (Figure 4A) [103]. In their study, the coated platelet membranes facilitated binding between nanoparticles and neutrophils through the recognition of P-selectin (platelet) and PSGL-1 (neutrophil). The internalized nanoconstructs then released piceatannol, which alleviated neutrophils’ infiltrations in the cerebral ischemic regions (Figure 4B). As a result, PTNPs significantly decreased the infarct volume of mice by approximately 26.2% compared with the group treated with PLGA nanoparticles containing piceatannol and superparamagnetic iron oxide.

Overall, platelet membrane coated nanoparticles have been extensively researched for thrombus, injured vessels and immune cell targeting to achieve higher on-target payload delivery and lower off-target side effects.

#### 4.2.3. Macrophage Membrane

Macrophages are specialized cells responsible for the detection, phagocytosis and destruction of “invaders”. Recent reviews have discovered the central role of macrophages in various cardiovascular-related processes including atherosclerotic coronary artery, post myocardial infarction remodeling and cardiac regeneration [104,105]. The macrophage membrane coating strategy is extensively applied to elevate tumor targeting ability through the driving force of CCR2-CCL2 axis, which could also be used to target inflammatory lesion sites [106,107].

Inspired by this, Gao et al. successfully utilized macrophage-derived membranes to coat nanoparticles and achieved enhanced therapeutic efficacy in atherosclerosis treatment [108]. In their study, they produced a ROS-responsive inner core to achieve burst release of the drug. The macrophage membrane coating strategy not only facilitates nanoparticles’ evasion of the monocyte phagocyte system (MPS) but also helps targeted delivery to the lesion, where the inner drug is rapidly released due to the oxidative environment of atherosclerosis. In addition, macrophage membranes can sequester proinflammatory cytokines, which could restrict local inflammation. A combination of pharmacotherapy and inflammatory cytokine clearance can significantly improve the therapeutic efficacy against atherosclerosis. Utilizing macrophage membranes’ intrinsic tendency toward atherosclerotic plaque, Wang et al. similarly constructed a macrophage membrane coated PLGA nanoplatform with anti-inflammation agent Rapamycin (RAP) loaded [109]. As a result, such nanoplatform exhibited outstanding therapeutic efficacy, in which lipid deposition in plaques were reduced from 36.45% to 17.41%, which was superior to free RAP group (from 36.45% to 31.54%).

Additionally, applying cytokine neutralization strategy, Xue et al. innovatively constructed a macrophage membrane enveloped NPs encapsulating anti-myocardial infarction (MI) agent miR-199a-3 (MMNP_miR199a-3p_) to manage MI [110]. In the study, they bioengineered macrophages to achieve enhanced expression of IL-1βR, IL-6R and TNF-αR. As a result, MMNP_miR199a-3p_ exhibits efficient uptake by myocardial cells and effective inhibition of inflammatory response through inflammatory cytokines neutralization.

Conclusively, inflammatory lesion site targeting ability and cytokine neutralization capacity are the most common usage of macrophage membrane in treating CVDs.

#### 4.2.4. Neutrophil Membrane

As mentioned above, neutrophils can sense and move to the inflammatory body site. Such inflammatory chemotaxis capability has been exploited to enhance drug on-target delivery. For example, Xue et al. achieved effective delivery of paclitaxel (PTX)-contained liposomes to the inflamed post-resection lesion sites mediated by neutrophils, resulting in suppressed glioma reoccurrence [111]. In addition, the feasibility of utilizing neutrophil-derived membranes to target the inflamed lesion has also been proven in rheumatoid arthritis [112].

At the inflamed lesion site, neutrophils are activated and subsequently release reactive oxygen species, bioactive lipid mediators and neutrophils extracellular traps (NETs), which induce inflammatory damage. Many cardiovascular therapies are based on targeting neutrophils, either through blocking neutrophils’ infiltration or preventing NET-driven inflammation [39].

Inspired by this, Dong et al. developed neutrophil membrane-derived nanovesicles containing Resolvin D2 (RvD2), an anti-inflammation agent, to treat postischemic stroke brain injury through inhibiting endothelial activation, cytokine release and the infiltration of neutrophils into the cerebral ischemic lesion [113].

**Figure 5:**
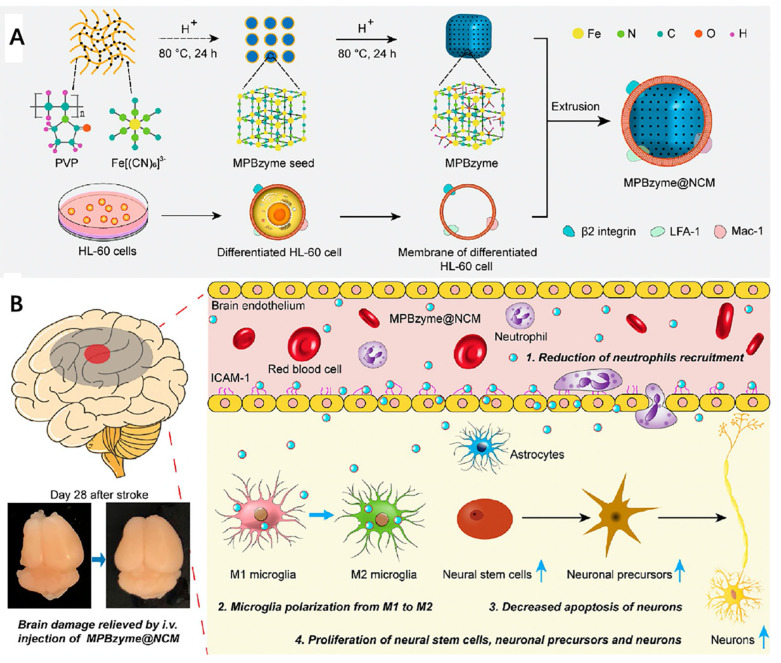
(**A**) Schematic structure of neutrophil-mimic membrane coated mesoporous Prussian blue nanozyme (MPBzyme@NCM); (**B**) Schematic diagram of the therapeutic mechanism. Combination of reduction of neutrophils’ recruitment, microglia polarization from M1 to M2, decreased apoptosis of neurons and proliferation of neural stem cells, neuronal precursors and neurons explain the therapeutic efficacy of MPBzyme@NCM. Reproduced with permission [114]. Copyright 2021, American Chemical Society.

In addition, Feng et al. also successfully constructed a neutrophil-mimic membrane coated mesoporous Prussian blue nanozyme (MPBzyme@NCM) (Figure 5A) [114]. In their study, MPBzyme@NCM exhibits active targeting ability to the inflamed brain microvascular endothelial cells, where nanoparticles are then phagocytosed by microglia and subsequently scavenge ROS through MPBzyme. As a result, this nanoplatform can promote microglia polarizing to M2 and reduce the neutrophils recruitment (Figure 5B), exhibiting prospective therapeutic efficacy against ischemic stroke.

In conclusion, the mainstream usage of neutrophil membrane against CVDs still focused on its inflammatory-lesion targeting ability. Further research is needed to explore other biomedical applications of neutrophil-derived membranes, such as cytokine neutralization.

#### 4.2.5. Stem Cell Membrane

The ischemic tissue-directed homing ability of mesenchymal stem cells has been reported. This process is mediated by the interaction of chemokine receptors on the surface of neural stem cells (NSCs) and their ligands enriched in the ischemic microenvironment such as SDF-1/CXCR4 axis [115,116]. Bose et al. first bioengineered human adipose-derived stem cells (hASCs) through mRNA vector transduction to overexpress CXCR4 [117]. This engineered stem cell membranes were then coated on the PLGA nanoparticles with vascular endothelial growth factor (VEGF) loaded to achieve better endothelial cell barrier penetration and increase retention time in ischemic tissues. As a result, this nanoplatform significantly improved therapeutic outcomes, achieving a lower risk of limb loss (17%) compared with the untreated group (83%).

To further improve the targeting ability, Kim et al. recently developed mesenchymal stem cell (MSC)-derived magnetic extracellular nanovesicles to treat ischemic stroke [118]. In their study, iron oxide nanoparticles were first co-incubated with MSC to elevate its expression of therapeutic growth factor. The whole MSCs were then extruded through serial membrane filters and purified to obtain magnetic nanovesicles (MNV). Overall, MNV enhanced lesion targeting ability through magnetic attraction and improved therapeutic efficacy against ischemic stroke.

## 5. Conclusions and Prospects

Ever since RBC membrane coating technology was first reported, countless investigations have been implemented to explore its therapeutic and diagnostic potential, for example in cancer therapy [119,120]. As mentioned in this review, various cell membranes are applied to prolong circulation time and endow nanoparticles with active targeting properties and inflammatory cytokines neutralization activity to treat CVDs (related studies have been integrated in Table 3).

However, we still lack a basic understanding of complex cell membrane properties. An inappropriate selection of blood type induces hemolysis during blood transfusion, which is attributed to the activation of the host immune system [121]. Therefore, autologous cells should be taken into prior consideration. Current studies on CMCNPs’ biomedical applications remain at the laboratory research stage, as most studies have been carried out in mice. With no current clinical usage of CMCNPs, investigations on their biosafety need to be implemented to accelerate their clinical translation.

Apart from biosafety issues, comprehensive studies on pharmacokinetics and cellular uptake of small molecule loaded CMCNPs need to be implemented. As various cell membranes and inner nanoparticles are involved in the synthetic procedure of CMCNPs, the impacts of membrane types and inner nanoparticles’ physical and chemical properties on the drug release ability of small molecules and cellular uptake of CMCNPs should be investigated extensively to facilitate their clinical translation.

The feasibility of large-scale production of CMCNPs is another barrier hindering their clinical translation. As aforementioned, membrane extrusion and sonication baths are two major routes to synthesize CMCNPs. However, these two approaches have distinctive strengths and weakness. For example, membrane extrusion is characterized with high uniformity but low-production efficiency, while the sonication method has contrary results. A better synthetic route is needed to overcome this obstacle.

**Table 3:** Cell membrane coated nanomedicine applied in treating cardiovascular diseases.

Target Disease	Structure (Membrane/Inner Core)	Membrane Source	Efficacy	Ref.
Atherosclerosis	RBC/PLGA	C57BL/6 Mice	(1) Enhanced accumulation in atherosclerotic plaques (2) Higher drug on-target release	[94]
	Platelet/UCNP	Healthy ApoE^−/-^ Mice	(1) Specific targeting to foam cells (2) Photodynamic therapy induced apoptosis and regulated lipid metabolism	[99]
	Platelet/PLGA	Human Type O^−^ Blood	(1) Avoid severe systematic toxicity of rapamycin (2) Enhanced 4.98-fold greater radiant efficiency than control nanoparticle group	[122]
	Macrophage/ROS-responsive core	RAW264.7 Cells	(1) Avoid immune clearance (2) ROS-responsive drug release (3) Inflammatory cytokine sequestration	[108]
	Macrophage/PLGA	RAW264.7 Cells	(1) Effectively inhibit phagocytosis by macrophages (2) Target and accumulate in atherosclerotic lesion	[109]
Thrombus	RBC/Janus-type NPs	Balb/c, Male	(1) Achieve movement through self-thermophoresis effect (2) RBC Membrane facilitate efficient movement in relevant biological environment	[95]
	Platelet/PLGA	ICR Mice	(1) Affinity between platelet membrane and thrombus (2) Lower the risks of adverse effect on the function of coagulation system	[97]
	Platelet/H_2_O_2_-degradable NPs	Human Type O^−^ Blood	(1) Thrombus homing ability of platelet membrane (2) H_2_O_2_ scavenging ability of inner polymer core (3) H_2_O_2_ responsive drug release ability	[98]
Ischemic myocardium	RBC/Mesoporous iron NPs	Male Sprague Dawley Rats	(1) Excellent biocompatibility (2) Extended circulatory time (3) Controlled-release of H_2_S	[123]
	Macrophage/miR_199a-3p_	RAW264.7 Cells	(1) Inflammatory cytokine sequestration (2) Gene delivery	[110]
	Platelet/IONP	C57BL/6 Mice	(1) Specific targeting to inflammatory neutrophils (2) Alleviate infiltrations of neutrophils into hypoxic lesion (3) Disease stage monitoring and nanoparticles localization through MRI	[103]
Ischemic stroke	Platelet/γ-Fe_2_O_3_ magnetic NPs	Blood Center	(1) Combination targeting ability of platelet membrane and magnetic forces (2) Disease stage monitoring and nanoparticles localization through MRI	[124]
	Neutrophil/MPBzyme	HL-60 Cells	(1) Reduction of neutrophils’ recruitment (2) Microglia polarization from M1 to M2 (3) Decreased apoptosis of neurons (4) Facilitate neuronal cells proliferation	[114]
	Stem cell/PLGA	C57BL/6 Mice	(1) Specific targeting ability toward ischemic microenvironment via stem cell membrane coating (2) Significantly augmented the efficacy of glyburide, an antiedema agent, for stroke treatment	[125]
Hindlimb ischemia	Stem cell/PLGA	Patients	(1) Bioengineered stem cell membrane coating for improved ischemic lesion targeting ability (2) Avoid macrophages phagocytosis	[117]
	Stem cell/IONP	Human Bone Marrow	(1) Stem cell preincubated with IONPs to elevate expression of therapeutic factors (2) Magnetic navigation improved the ischemic-lesion targeting	[118]

On the other hand, challenges and opportunities coexist in this field. Many alternative membrane options are under investigation, such as cancer cells [66,87], bacteria [89,90] and even hybrid cell membrane vesicles [89,126]. For example, Gu et al. successfully fabricated “Nano-Ag@erythrosome” nanocomplexs by fusing red blood cell membranes and cancer cell membranes [127]. Such nanocomplexes can effectively target the spleen due to the property of senescent red blood cell membrane, and induce antigen presentation on antigen presenting cells (APCs) to activate an antitumor immune response. Further research may well grasp this strategy and further modify this method by adjusting the adding ratio of different membrane sources to achieve elevated targeting efficiency and improved therapeutic efficacy against cardiovascular diseases [128].

Furthermore, recent research has reported a selective organ targeting (SORT) system for specific tissue mRNA delivery by introducing different SORT lipid into lipid nanoparticles through a bottom-up chemical synthesis method [129]. A combination of bottom-up and top-down CMCNPs synthesizing strategies exhibits great promise in extending their biofunctions and broadening their biomedical applications. Apart from this, lipid insertion, metabolic substrates engineering and genetic modification are other conventional membrane engineering methods, which also exhibit promise to extend the biofunctions of the derived cell membranes and have already been summarized extensively elsewhere [130,131,132,133]. In general, these abovementioned measurements, which aim to expand the biomedical application of CMCNPs, are all based on membrane engineering. The appropriate combination of inner functional nanoparticles with selected and modified outer-membranes will significantly enhance the therapeutic efficacies of CMCNPs. Overall, more innovative strategies will be explored in the future to unlock a new stage for cell membrane coated nanoparticles to treat cardiovascular diseases.

## Figures and Tables

**Table 1:** Common inner particles applied in core–membrane strategy.

Core Particle	Properties	Application	Ref.
PLGA	Biocompatibility and biodegradability Easy manipulation	Drug carrier	[58]
Liposome	Hydrophobic and hydrophilic drug delivery Sustained drug release	Drug carrier	[62,63]
MSN	Tunable pore size High pore volume	Drug carrier	[65,66]
UCNP	Convert NIR into visible light	Deep tissue imaging	[76]
Gold NPs	Photothermal effect	Photothermal therapy	[64]
IONP	Magnetic property	MRI Magnetic targeting	[67,68]
BP Nanosheet	Photothermal conversion	Photothermal therapy	[69,70,71]

**Table 2:** Main usage of several common cell membranes.

Membrane Source	Properties	Application	Ref.
Red blood cell	Immune evasion	Prolong circulation	[82]
Platelet	Selective targeting to injured tissue Adherence to inflammatory neutrophil	Targeting cancer metastasis Targeting vascular injury	[83]
Macrophage	Immune evasion Cytokine sequestration	Inflammatory site targeting Anti-inflammation	[84,85]
Neutrophil	Selective targeting to inflammatory tissue	Inflammatory site targeting	[86]
Cancer cell	Tumor targeting Antigen delivery	Homotypic targeting Cancer vaccine	[87]
Stem cell	Penetration across the endothelium	Tumor targeting Inflammatory migratory	[88]
Bacterium	Elicit immune response Anti-adhesion	Cancer immune therapy Bacterial infection	[89,90]

## Data Availability

Not applicable.

## Sample Availability

Samples of the compounds are not available from the authors.
